# Mutation accumulation and fitness effects in hybridogenetic populations: a comparison to sexual and asexual systems

**DOI:** 10.1186/1471-2148-7-80

**Published:** 2007-05-21

**Authors:** Christian Som, Homayoun C Bagheri, Heinz-Ulrich Reyer

**Affiliations:** 1 Zoological Institute, University of Zürich, Winterthurerstrasse 190, CH-8057 Zürich, Switzerland; 2 World Wide Fund for Nature (WWF), Hohlstrasse 110, CH-8010 Zürich, Switzerland

## Abstract

**Background:**

Female only unisexual vertebrates that reproduce by hybridogenesis show an unusual genetic composition. They are of hybrid origin but show no recombination between the genomes of their parental species. Instead, the paternal genome is discarded from the germline prior to meiosis, and gametes (eggs only) contain solely unrecombined maternal genomes. Hence hybridogens only transmit maternally inherited mutations. Hybridity is restored each generation by backcrossing with males of the sexual parental species whose genome was eliminated. In contrast, recombining sexual species propagate an intermixed pool of mutations derived from the maternal and paternal parts of the genome. If mutation rates are lower in female gametes than males, it raises the possibility for lower mutation accumulation in a hybridogenetic population, and consequently, higher population fitness than its sexual counterpart.

**Results:**

We show through Monte-Carlo simulations that at higher male to female mutation ratios, and sufficiently large population sizes, hybridogenetic populations can carry a lower mutation load than sexual species. This effect is more pronounced with synergistic forms of epistasis. Mutations accumulate faster on the sexual part of the genome, and with the purifying effects of epistasis, it makes it more difficult for mutations to be transmitted on the clonal part of the genome. In smaller populations, the same mechanism reduces the speed of Muller's Ratchet and the number of fixed mutations compared to similar asexual species.

**Conclusion:**

Since mutation accumulation can be less pronounced in hybridogenetic populations, the question arises why hybridogenetic organisms are so scarce compared to sexual species. In considering this, it is likely that comparison of population fitnesses is not sufficient. Despite competition with the sexual parental species, hybrid populations are dependent on the maintenance of – and contact with – their sexual counterpart. Other problems may involve too little genetic diversity to respond to changing environments and problems in becoming hybridogenetic (e.g. disruption of meiosis and subsequent infertility or sterility). Yet, lower mutation accumulation in hybridogenetic populations opens the possibility that hybridogenetic species can develop into new sexual species once recombination is re-established and reproductive isolation from sexual ancestors has occurred.

## Background

Finding explanations for the evolution and maintenance of sexual reproduction is a long-running research problem in evolutionary biology. A large number of scenarios and models have been developed under which either sexual or asexual reproduction could have advantages or disadvantages [1]. In the context of this ongoing search, unisexual (all-female) vertebrates are of great interest, because they put popular explanations like Muller's ratchet [2,3], mutational deterministic hypothesis [4] and the Red Queen [5-7] to a test. All unisexual vertebrates are of interspecific hybrid origin [8]. They therefore have similar genome sizes (with the exception of polyploid species), are exposed to similar environments as their sexual parental species and often directly compete against them. This allows for the comparison of the two reproductive modes under similar ecological conditions.

**Figure 1:**
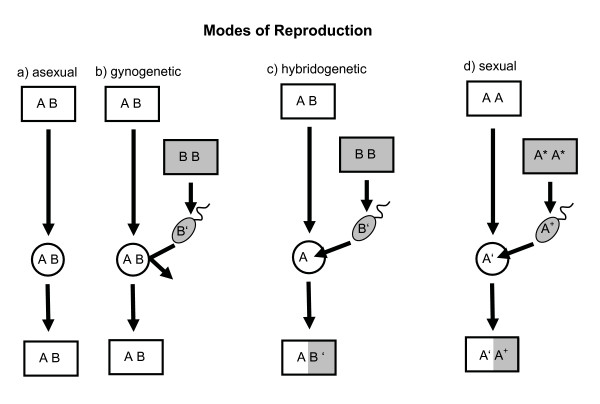
**Three modes of reproduction in all-female species of hybrid origin (a-c) compared to bi-sexual reproduction in true species (d)**. Boxes represent diploid individuals, circles and ellipses represent eggs and sperm, respectively. Letters in boxes depict genomes of two different species (A and B in Figure 1a–c) or different sexes within a species (A and A* in Figure 1d). Superscripts (' and ^+^) on letters in gametes indicate that, due to recombination, these gametes contain a unique combination of genes, whereas gametes without superscripted letters contain clonal genomes. As a result of the different reproductive modes the offspring are genetically either identical to their mother (AB in Figure 1a, b), highly variable (A'A^+^ in Figure 1d) or intermediate with one clonal and one recombined genome (AB' in Figure 1c). White = females, grey = males. For further explanations see Introduction.

Whereas the theories of mutation accumulation and selection against deleterious mutations are well established for asexual and sexual species, they are not for unisexual vertebrates that reproduce by hybridogenesis, a reproductive mode that is intermediate between sexual and asexual reproduction. Figure 1 illustrates this intermediate position for the typical vertebrate case. Parthenogenesis (Figure 1a) and gynogenesis (Figure 1b) both represent asexual reproduction, where offspring arise clonally from diploid eggs of an all-female species (AB) that in the past originated from hybridization between two sexual species (AA and BB). In parthenogenesis the eggs develop by themselves, whereas in gynogenesis their development must be triggered by sperm from a male of one of the hybrid's ancestral parental species (genotype BB in Figure 1). Hence, gynogenetic females must mate, but the male's genome (B') does not show up in the offspring. Hybridogenesis (Figure 1c) resembles gynogenesis in that females of hybrid origin (AB) need males for successful reproduction. In contrast to gynogens, however, hybridogenetic females discard the ancestral paternal genome prior to meiosis (B in Figure 1c), produce haploid eggs with an unrecombined maternal genome (A) and then restore diploidy (and hybridity) in their offspring by backcrossing with males of the parental species whose genome was discarded. In this inclusion of the paternal genome they resemble true bi-sexual species (Figure 1d). Thus, the genome of a hybridogen is "hemiclonal", consisting of a clonally inherited maternal part and a sexually inherited paternal part, with no recombination between them [9].

The long time evolutionary perspectives of such hybridogenetic unisexuals have been questioned by several authors [e.g. [10-12]] based on the argument that clonal inheritance of a part of their genome exposes them to the same perils of reduced genetic diversity as parthenogenetic or gynogenetic species. However, hybridogenetic reproduction has not been modelled yet in terms of deleterious mutation accumulation dynamics and susceptibility to drift effects. Given the low number of unisexual vertebrates (some 70 species; 10, 13], one may ask: why should we even care? In 1969, Schultz [9] proposed that hybridogenesis may act as a transition state in the formation of new species, and Vrijenhoek [11] found some evidence for such an event in a sexual species of *Poeciliopsis *with supposed hybridogenetic ancestry.

Such speciation events can only be successful if the newly arising species has not accumulated too many deleterious mutations during its hybridogenetic history. In evaluating the risk of mutation accumulation in hybridogens, as compared to other reproductive modes, one has to consider that parthenogenetic (including gynogenetic) and hybridogenetic vertebrates are all-female species (with the exception of hybridogenetic water frogs), whereas sexual species consist of males and females. This becomes important when mutation rates are sex-specific. Starting with Haldane [14,15] a number of studies have shown higher mutation rates in males than in females. Current reviews on male/female mutation rates (α) list ratios in the range from 1 to 10 for primates, rodents, birds and humans, but data are still very scarce [16-18]. The male bias has been interpreted as evidence that new mutations occur during DNA replication, as spermatogonia divide throughout the whole life of males whereas oogenesis in females is largely complete at birth. Therefore, it has been suggested that evolution is "male driven".

Since direct measures of sex specific mutations rates are difficult to obtain, Miyata *et al*. [16] proposed an indirect method of testing for sex differences in mutation rates, namely comparing the evolutionary rate of sex chromosomes and autosomes. An X chromosome has spent about 1/3 of its history in males, whereas autosomes spend about an equal time in both sexes, and Y chromosomes only occur in males. If male and female mutation rates are different, we expect different evolutionary rates for X and Y chromosomes and autosomes.

Redfield [19] modelled the effect of elevated male mutation rates on the mutational load in infinite sexual populations and compared the results with those from infinite diploid asexual populations. She showed that the cost of male mutations can easily exceed the benefit from recombination if populations are sufficiently large. Since hybridogenetic unisexuals are often in direct competition with their sexual parental species due to the forced coexistence, such systems allow testing the effects of sex-specific mutations rates on the relative success of sexual versus asexual reproduction. We therefore expanded Redfield's model for infinite populations and included hybridogenetic reproduction into the comparison. Furthermore, we developed Monte-Carlo simulations for finite sexual, diploid asexual and hybridogenetic populations of two different sizes (2000 and 200 individuals, respectively) to account for the effect of higher male mutation rates and stochastic events like drift effects and Muller's ratchet.

Details of the models used in this paper are described in the Methods section. All the three model populations investigated (infinite, 2000 and 200 individuals, respectively) share the following common features:

- Generations do not overlap

- Mating is random

- Males and females can mate several times

- Individuals accumulate new mutations between birth and reproduction. The distribution of these mutations follows a Poisson distribution with the mean of *U*

- All mutations have the same character of dominancy and the same effect on fitness, regardless of the locus where they occur. Hence they are assumed to be co-dominant.

- All populations (asexual, sexual and hybridogenetic) are diploid.

- A homozygous mutation, i.e. with 2 mutated alleles at the same locus, has the same fitness effect as 2 heterozygous loci bearing a mutated allele.

- Sexual individuals show Mendelian recombination

- Hybridogenetic individuals do not recombine

- All hybridogenetic populations (including the finite populations) live in sympatry with an infinite sexual population maintained in mutation-selection balance.

- Sympatric sexual and hybridogenetic populations show the same sex specific mutation rates and both populations show the same type of mutation interaction.

- New mutations in a hybridogen are equally likely to occur on the sexual or on the clonal part of the hybridogen's genome

**Figure 2:**
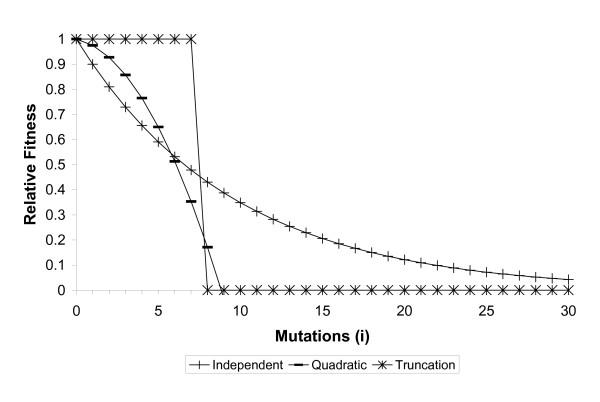
**Types of selection against mutations with variable degrees of synergistic epistasis (after Redfield, 1994)**. The vertical axis shows the relative fitness of an individual with *i *deleterious mutations compared to an individual with no deleterious mutations. Independent selection (no epistasis): relative fitness *W*_*i *_= 0.9^*i*^. Quadratic selection (medium epistasis): *W*_*i *_= 1-0.014i - 0.0112i^2 ^[38]. Truncation selection (high epistasis): *W*_*i *_= 1 if *i *< = 7, else *W*_*i *_= 0.

All three reproduction modes (sexual, asexual and hybridogenetic) were compared under the same three population sizes (infinite, 2000 and 200 individuals) and the same three types of mutation interaction with different levels of epistasis: independent, quadratic and truncation selection (Figure 2).

## Results

**Figure 3:**
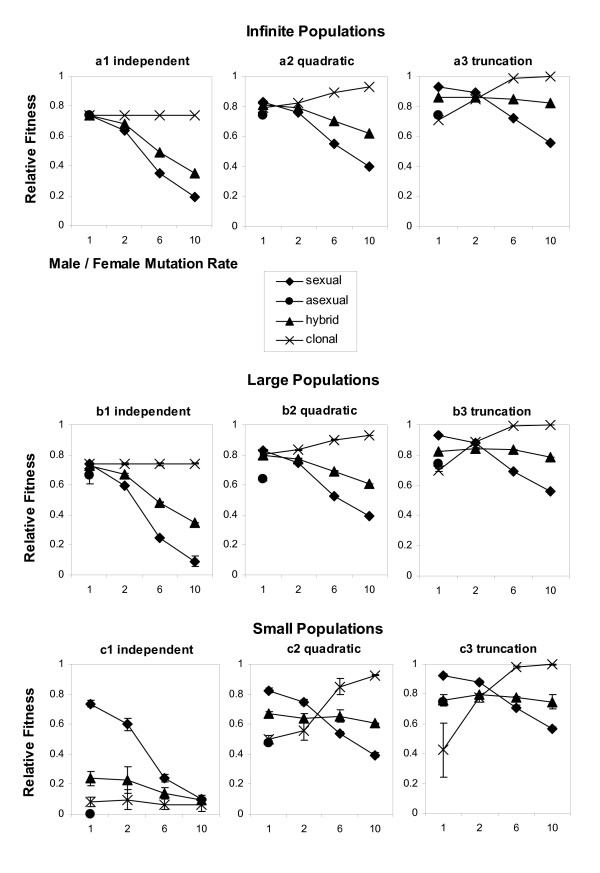
**Average population fitnesses after 8000 generations**. Fitness values are plotted in relation to population size, type of selection against mutations and the male to female mutation rate ratio. Vertical axes show the relative model population fitnesses compared to a mutation free population. Horizontal axes show the male to female mutation rate ratios α. Large populations consist of 2000 individuals and small populations of 200 individuals. The legend shows the type of reproduction. The term hybrid refers to hybridogenetic reproduction and the term clonal refers to the fitness of a hypothetical population with a pair of chromosomes derived only from the clonal part of the hybridogenetic genomes. For easier comparison, the fitness under asexual reproduction at α = 1 is indicated by a dashed line extending over the whole range of α values. Data points in Figure 2 parts b1-b3 and c1-c3 show the average of 10 runs per parameter set. Standard deviations are indicated on the data points but are often smaller than the symbols.

Since the results for large populations with 2000 individuals are almost identical to those for infinite populations (see Figures 3a and 3b), they will be presented together

### (i) Infinite and large populations

Figures 3a1–a3 and 3b1–b3 show the results of the test runs for the infinite and the large populations. The results correspond well with those of Kimura & Maruyama [20] and Redfield [19] who modeled the effects of epistasis on mutational load in relation to sexual and asexual reproduction. If mutations show no synergistic epistasis (independent selection, Figure 3a1 and 3b1) and male and female mutation rates are the same (α = 1), all reproductive modes perform equally. With increasing male to female mutation rate ratio α, asexual reproduction becomes favorable compared to sexual reproduction. Hybridogenetic populations show an intermediate mutation load between asexual and sexual populations, whereas the clonally transmitted genomes of hybridogens accumulate the same number of deleterious mutations as the genomes of an infinite asexual population (W¯
 MathType@MTEF@5@5@+=feaafiart1ev1aaatCvAUfKttLearuWrP9MDH5MBPbIqV92AaeXatLxBI9gBaebbnrfifHhDYfgasaacH8akY=wiFfYdH8Gipec8Eeeu0xXdbba9frFj0=OqFfea0dXdd9vqai=hGuQ8kuc9pgc9s8qqaq=dirpe0xb9q8qiLsFr0=vr0=vr0dc8meaabaqaciaacaGaaeqabaqabeGadaaakeaadaqdaaqaaiabdEfaxbaaaaa@2DF4@ = 0.74 for all α). Under independent selection, asexual reproduction was the only reproductive mode where large finite populations would suffer from fixations of mutations. In four out of ten runs the model population ended with one fixed mutation at a locus and in three cases with two fixed mutations

With both the quadratic and truncation forms of synergistic epistasis (Figures 3a2, a3, b2, b3), when α is approximately larger than 2, the overall ordering of fitness relationships between sexuals and asexuals remains unaltered. However, as α approaches a value of 1, we have a reversal, whereby sexual reproduction shows an advantage over hybridogenetic and asexual reproduction. This advantage becomes larger with stronger synergistic epistasis (truncation selection, Figures 3a3, b3); an observation that is consistent with the mutational deterministic hypothesis [20,21]. With α = 1 and high levels of synergistic epistasis, recombination is more effective in reducing the mutation load; consequently, sexual reproduction is more advantageous than asexual reproduction [19]. Note that for α = 1, the advantage of sexual reproduction would be also more pronounced if mutation rates *U* were to be increased [20,21].

For the epistatic cases with α = 1, hybridogens show behavior that is generally intermediate between the asexuals and the sexuals. This is because they have an advantage over the asexuals, since the sexual part of their genome – derived form the parental species – has been subject to the purifying effects of recombination. Meanwhile, as α becomes larger, they do better than the sexuals because the clonal half of their genome is not subject to the increased accumulation of mutations incurred in their sexual counterpart.

Furthermore, an interesting – and somewhat counterintuitive – aspect of the simulation results is the observation that the mutational load on the clonally transmitted part of the hybrid genome does not increase, but rather is reduced as α increases. In effect, with truncation selection, hybridogenetic populations show a higher average fitness than asexual populations for all tested α values, and a higher fitness then sexual populations for α ≥ 2 The more the paternal genome is loaded with mutations relative to the maternal genome, the more truncation selection prevents the accumulation of mutations on the clonally transmitted part of the hybridogenetic genome. At high α values, the clonal part of the genome is virtually free of deleterious mutations.

Under asexual reproduction, the nonexistent selection against the first few mutations per individual led to the fixation of the maximum number of 7 allowed mutations in the finite populations, whereas under sexual reproduction no fixations occurred. With hybridogenetic reproduction, the number of fixed mutations decreases from 2 at α = 1, through 1 at α = 2 to 0 at higher α values.

### (ii) Small populations

Small populations (200 individuals) are prone to accumulate higher numbers of mutations due to increased drift effects. This effect can be seen well in the case of independent mutational effects on fitness (Figure 3c1). All asexual test populations reached the maximum number of mutations the computer simulation could handle (100), and the average population fitness plunged to a level which would not allow persistence anymore (W¯
 MathType@MTEF@5@5@+=feaafiart1ev1aaatCvAUfKttLearuWrP9MDH5MBPbIqV92AaeXatLxBI9gBaebbnrfifHhDYfgasaacH8akY=wiFfYdH8Gipec8Eeeu0xXdbba9frFj0=OqFfea0dXdd9vqai=hGuQ8kuc9pgc9s8qqaq=dirpe0xb9q8qiLsFr0=vr0=vr0dc8meaabaqaciaacaGaaeqabaqabeGadaaakeaadaqdaaqaaiabdEfaxbaaaaa@2DF4@ = 0.00003). Sexual reproduction successfully prevented the fixation of mutations with mean population fitnesses in the range of the larger and infinite populations. In hybridogenetically reproducing populations, the clonally transmitted part of the hybrid genomes suffered from substantial fixation of mutations in the small populations (min. 8, max 14 fixed mutations, av. 10.8). The resulting low clonal fitness leads to a strongly diminished average fitness in hybridogens as well.

Under quadratic selection (Figure 3c2), mutation accumulation and fixation in small asexually reproducing populations is not as serious as under independent selection but still worse than with the other two reproductive modes (W¯
 MathType@MTEF@5@5@+=feaafiart1ev1aaatCvAUfKttLearuWrP9MDH5MBPbIqV92AaeXatLxBI9gBaebbnrfifHhDYfgasaacH8akY=wiFfYdH8Gipec8Eeeu0xXdbba9frFj0=OqFfea0dXdd9vqai=hGuQ8kuc9pgc9s8qqaq=dirpe0xb9q8qiLsFr0=vr0=vr0dc8meaabaqaciaacaGaaeqabaqabeGadaaakeaadaqdaaqaaiabdEfaxbaaaaa@2DF4@ = 0.48, 5 fixed mutations in all test populations). Sexual populations seem to be largely resistant to drift effects, since even at a size of 200 they do not differ much from infinite populations in their average fitness, and no mutations got fixed within 8000 generations. Susceptibility of hybridogenetic populations to drift effects varies with male to female mutation rates: averaged over the ten model populations the number of fixed mutations decreased from 2.0 and 1.9 at α = 1 and α = 2, respectively, through 0.4 (α = 6) to 0 (α = 10). Despite the occasional fixation of mutations, small hybridogenetic populations showed a higher average fitness at high α values than small sexual populations. The clonally transmitted hybrid genomes showed reduced fitness at α = 1, 2 due to the fixed mutations; but at higher male to female mutation rate ratios, the strong selection against new mutations prevented these genomes from accumulating and fixing mutations and, hence, resulted in high fitness.

Under truncation selection, the nonexistent selection against low numbers of deleterious mutations led to the accumulation and fixation of the maximum allowed number of 7 mutations in all asexual model populations; but nevertheless, the average mean population fitness did not degrade compared to the large and the infinite populations, because the fixed mutations do not cause a decline in fitness. No fixation of mutations occurred in the sexual populations, and again population size had only little effect on the average population fitness (compare Figure 3a3–3c3). Although hybridogens show similar levels of fitness as asexuals for all α values, they fix fewer mutations on their clonal genomes than asexuals do for all tested α > 1, namely on average 2.0, 1.0 and 0.3 for α = 2, 6 and 10, respectively, compared to 7 in asexuals.).

### (iii) Speed of Muller's ratchet

**Figure 4:**
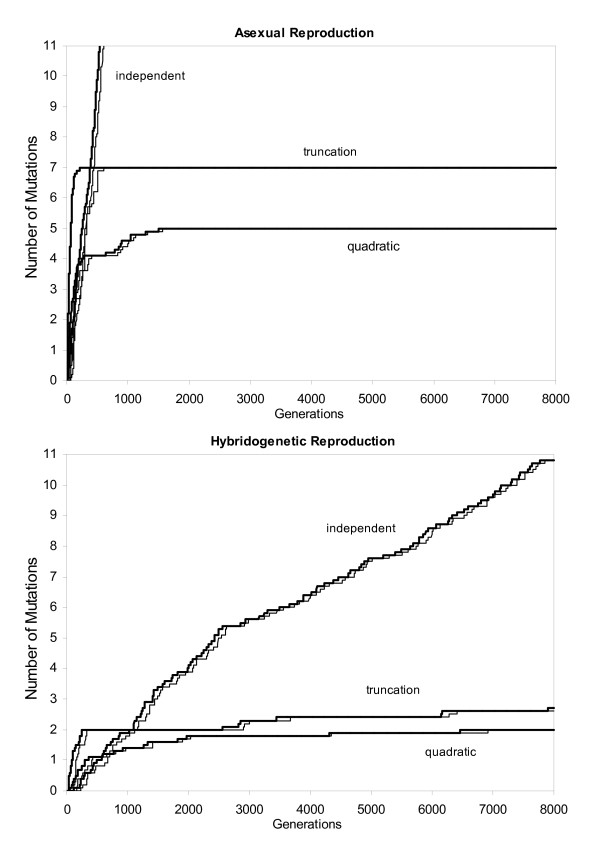
**The speed of Muller's ratchet in small (200 individuals) asexual and hybridogenetic populations**. The vertical axis shows the number of lost classes respectively the number of fixed mutations. Both graphs show the average of 10 runs per reproductive mode. *U *was set to 0.3 for both reproductive modes and α = 1 for hybridogenetic reproduction to allow a comparison between asexual and hybridogenetic reproduction. Thicker lines indicate the losses of mutation classes whereas thinner lines indicate the number of fixed mutations in the population.

Since we recorded the losses of least loaded classes and the fixation of mutations during the model runs, this allowed us to determine the speed of Muller's ratchet. Finite populations of the larger size (2000 individuals) only occasionally showed fixations of mutations (results not shown here) but in smaller populations, fixations occurred frequently enough to allow a comparison of the speed of Muller's ratchet between asexual reproduction and hybridogenetic reproduction with α = 1. In this comparison, both model populations had the same genome size and genomic mutation rate. Figure 4 shows the average of 10 test runs per reproductive mode and type of mutation interaction. The fixation of mutations followed the loss of the least loaded classes closely, regardless of the type of reproduction, which confirms the findings of Charlesworth & Charlesworth [3]. Under hybridogenetic reproduction, Muller's ratchet not only clicks significantly slower than under asexual reproduction; the level of fixed mutations at which the ratchet would come to a near halt (quadratic and truncation interaction) is also lower.

## Discussion

Longtime evolutionary perspectives for hybridogenetically reproducing organisms often have been considered as not being very promising [e.g. [10,22]] but knowledge of the dynamics of deleterious mutation accumulation and selection in hybridogens has been limited so far. Since one half of the hybridogens' genome is passed on without recombination from generation to generation, it is understandable that the mechanisms of mutation accumulation have been expected to operate in a similar manner as in asexual populations [e.g. [10]]. However, this expectation does not necessarily hold. As the results in this study indicate, the dynamics of mutation accumulation in hybridogens can differ from the ones in asexual populations; both in terms of the speed of mutation accumulation and (depending on the type of interaction effects between mutations)  the total number of deleterious mutations.

The fitness effects of deleterious mutation accumulation in hybridogens are not simply at an intermediate level between sexual and asexual populations. The latter comparison depends on the ratio of male to female mutation rates α. If α = 1 in a large population with synergistic epistasis, conventional expectations hold and the average fitness of a hybridogenetic population is indeed intermediate between sexuals and asexuals. This intermediate position is due to the property that mutation accumulation is slower within the chromosomes derived from the sexually reproducing parental species (where mutations are more effectively purified due to synergistic epistasis and recombination). However, the situation starts to change as α becomes larger than a value of one. In the latter case, sexually reproducing species not only face the often cited twofold costs of reproduction [23], but also suffer from a higher average mutation rate than comparable all female asexual species (given that males would have a higher mutation rate than females [19]). Since hybridogenetic organisms do not recombine, the mutations originating on the sexual parent's gamete are not passed on to the next generation. Whereas gametes of sexuals contain, on average, half of the paternally inherited, half of the maternally inherited mutations plus half of the newly acquired mutations, the female gametes of hybridogens contain only the maternally inherited mutations plus the newly acquired mutations that occurred on the clonally transmitted part of the genome.

To understand the simulation results presented here, it is necessary not only to consider the mechanistic differences in modes of genetic transmission, as represented by the three reproductive systems discussed here. It is also necessary to understand the interplay of population dynamics with these mechanisms. This becomes apparent when one considers mutation accumulation in the clonal part of the hybrid genome. As seen in the cases with quadratic and truncation selection (Figure 3), as α becomes larger, mutation accumulation goes towards zero in the clonal part of the hybrid genome (fitness = 1).

This is not because mutations do not occur on the clonal part, but rather that they accumulate much faster on the sexual part of the genome, and hence the quadratic and truncation selection effects allow for no more mutations to accumulate on the whole genome. Selection is too effective in purging the "late arriving" mutants on the clonal part of the genome. In other words, if all mutations that a hybridogen carries affect fitness, regardless whether they reside on the sexually or clonally transmitted part of the genome, selection against all newly acquired mutations is strong if mutations interact synergistically, because of the "borrowed" high mutational load on the paternal genome. Since only the maternal genome can be transmitted (which has been under strong selection against new mutations), the clonally transmitted part of the genomes of hybridogenetic organisms stays remarkably (although not totally, see [24]) free of deleterious mutations. The higher the male to female mutation rate ratio in the sexual parent, the more pronounced is this "shielding effect" in hybridogens. In large populations with truncation selection, this effect can be strong enough that even at an α approaching 10, the fitness of the hybridogenetic population can be higher than its asexual counterpart (Figures 33a3, 3b3).

A challenge for the persistence of asexually and hemiclonally reproducing organisms are drift effects in small or fragmented populations [25]. The comparison of the speed of Muller's ratchet between asexual and hybridogenetic populations (Figure 4) showed an effective reduction in the ratchet speed for hybridogenetic populations. This advantage originates from the model properties that only half of a hybridogen's new mutations occur on its clonally transmitted part and, thus, can be transmitted to the next generation, whereas in an asexual organism all novel mutations are transmitted to the offspring. In this model, fixation of mutations followed the loss of the least loaded fitness classes in asexual populations (as previously shown by Charlesworth & Charlesworth [3]) and hybridogenetic populations (as shown here).

Although population size had little influence on the mutational load and on the number of fixed alleles in the size-limited populations with epistatic mutation interaction, hybridogenetically reproducing populations were generally less prone to the accumulation of deleterious mutations than asexual populations. The selective forces against deleterious mutations seemed to be sufficiently effective to prevent a substantial drop in average population fitness (especially at high α values), as the mutation rate on the clonally transmitted genome is reduced relative to that on the sexual genome.

Even in quite small populations, sexually reproducing organisms are at a disadvantage compared to all female asexual populations if the male to female mutation rate ratio is high, at least under quadratic and truncation selection (Figure 32c2–c3). This result could be due to our choice of the low rate of 0.3 deleterious mutations per female and generation in the asexual populations. However, the effective values of *U *are still debated [e.g. [26,27]], and the value chosen for our simulations seems to be somewhere in the middle of the reported ranges. Furthermore, our *U*-value of 0.3 allows a direct comparison with the results of Redfield [19]. At high levels of α, small hybridogenetic populations accumulate fewer mutations than small populations of their sexual parent species if mutations interact synergistically. Since hybridogenetic populations are better protected against the accumulation and fixation of deleterious mutations than asexual populations, and at the same time, are less affected by the negative impacts of a higher deleterious mutation rates in males, hybridogenetically reproducing organisms perform better than asexual and sexual organisms at reduced population sizes under quadratic and truncation selection if α > 2. This result is quite interesting as this is the area, where the average *U *is between 1.05 and 1.65, and, therefore, in the range where sexual reproduction becomes favorable over asexual reproduction in mutational deterministic models [21,28].

## Conclusion

The simulation results in this work indicate that with male to female mutation rates higher than one (α > 1), hybridogenetic populations can be less prone to the accumulation of deleterious mutations, and hence have a higher population fitness than their sexual counterpart. As α becomes larger, mutation accumulation in the part of the genome derived form the sexual species is faster, and these mutations make it more difficult for new mutations to accumulate on the clonal part of the genome (which has a slower mutation rate). This effect becomes more pronounced with synergistic forms of epistasis (quadratic and truncation). Furthermore, in cases with epistasis and α = 1, although sexual populations have a higher fitness than the hybrids, the hybrids in turn do better than the asexuals, because mutations are purged more effectively in the sexually derived part of their genome.

These results lead to the question why hybridogenetic organisms are so scarce compared to sexual species. At the genetic level, one reason could be that, in the real world, mutation interactions are far more complicated and diverse than the ones used in this model [29,30]. If we assume that mutation interactions vary from negative through no to positive epistasis, with an average effect of no epistasis, sexual reproduction still acts best against the accumulation of mutations in very small populations (Figure 2c1). Also, in natural populations, neither selection nor dominance coefficients are constant values for all deleterious mutations. Another reason why hybridogens are scarce could be that the real obstacle is actually to achieve hybridogenetic reproduction [10]. In order to do so, meiosis has to be circumvented or disrupted which often leads to infertility or sterility [31].

Furthermore, one cannot consider the evolution of hybridogenesis by only looking at genetic mechanisms but has to include ecology as well. The survival of the hybrid population totally depends on the continued contact with the sexual parental population which serves as an indispensable sperm donor. Their sperm-dependence prohibits the hybridogens to occupy niches which are markedly different from the niche of the sexual parent and does not allow out-competing the parental species and leading it to extinction. Hence, hybridogenesis can only evolve and persist under ecological conditions that allow stable populations of both, the sexual parasite (i.e. the hybridogens) and their sexual hosts (i.e. the parental species). This ecological issue was not considered in the present work, but has been dealt with in previous publications [32-35]

Our result, that clonal or hemiclonal polymorphism can not be maintained with the used model framework, suggests that the often observed diversity in sympatric clones or hemiclones either originates from subsequent recruitments of new clones or hemiclones through repeated primary hybridization, or that diversity in clones is maintained through the occupancy of different microhabitats or "frozen niches" [36-39]

Although mutation accumulation is less of a threat to hybridogenetic populations (in comparison to sexuals and asexuals) under several of the conditions used in this model, there are other factors inherent to asexual or hybridogenetic reproduction which affect the long time success of such populations: the response to rapidly changing environments or to parasites is still best ensured through sexual reproduction [40,41]. But nevertheless, the results of this study indicate that, as suggested by Schultz [9], hybridogenetic species could in fact act as a transition state in the formation of new sexual species once recombination is reestablished and reproductive isolation from both sexual ancestors occurred. The surprisingly small accumulation of deleterious mutations on the clonally transmitted part of a hybridogen's genome under synergistic epistasis and differential sex specific mutation rates supports this as an option for new species formations.

## Methods

Here, we describe the models for infinite und finite populations, and how the respective simulations were implemented. The features common to all model populations have been mentioned in the Background section.

### (i) Modelling epistasis and fitness functions

In our simulations, we used three different types of selection against mutations, each represented by a fitness function with a specific form of gene interaction (after Redfield, 1995): namely independent, quadratic, and truncation interaction (Figure 2). The function for independent interactions represent additive effects (i.e. no epistasis), whereby if plotted on a logarithmic scale, the fitness function is linear. The quadratic function represents synergistic epistasis, whereby successive mutations have a larger relative effect on fitness. The truncation function is an extreme form of synergistic epistasis.

#### Independent effects (no interactions)

Let W_i _be the relative fitness (in terms of the number of gametes it is able to produce) of individuals with i mutations compared to an individual with no mutations. With independent fitness effects,

*W*_*i*_*= (1-s)*^*i*^,

where *s *is the selection coefficient for each new mutation. For our simulations, we chose an *s *of 0.1, so the relative fitness of an individual with i mutations is *W*_*i*_*= (0.9)*^*i*^

#### Quadratic effects (synergistic interactions)

Again, let W_i _be the relative fitness of individuals with i mutations compared to an individual with no mutations. Based on Kimura and Maruyama [20], and Redfield [19] the synergistic fitness function used is

*W*_*i *_= 1 - *h*_1_*i *- *h*_2 _*i*^2^,

where *h*_1 _and *h*_2 _are constants. The third term in this equation is the nonlinear term that produces the synergistic effects as *i *becomes larger. In our simulations, *h*_1 _= *0.014 *and *h*_2 _= *0.0112 *[38]. For values of i where *W*_*i *_≤ 0, we set the value of *W*_*i *_to zero.

#### Truncation selection (threshold dependent synergistic interactions)

Under truncation interaction, accumulated mutations do not have a negative effect on fitness until the number of mutations reaches a threshold. Beyond this threshold, fitness drops to zero, thus *W*_*i*_*= 1 *if *i *< = 7, else *W*_*i*_*= 0*.

### (ii) Simulations for infinite populations

#### Asexual populations

According to Kimura & Maruyama [20] the mutation load of an infinite asexual population can be determined by dividing the population into classes of individuals bearing the same number of deleterious mutations. The number of mutations that individuals acquire follows a Poisson-distribution with the mean of *U*, the genomic mutation rate per individual and generation. Hence the probability *P*(*j*) that an individual acquires *j *mutations is given by *(j) *= *U*^*j*^*e*^-*U*^*(j!)*^-1^. Let us assume that all individuals reproduce at the same time and that generations do not overlap and that there are no back mutations. The class of individuals with *i *mutations at the beginning of their lifespan originates from a set of parents who could be sequentially ordered into classes (*c*_0_, *c*_1_,..., *c*_*i*_), composed of parents with 0 to *i *mutations. Individuals in each parental class *c*_*i*-*j*_, would have to produce *j *mutations to produce progeny in the class *c*_*i*_. Hence, the number of individuals in class *c*_*i *_is the sum of the progeny from each class *c*_*i*-*j *_with *j *mutations, where 0 ≤ *j *≤ *i*. If *W*_*i *_is the relative fitness of individuals in the class with *i* mutations compared to the class with no mutations and fitness is expressed in the number of gametes an individual can produce, the frequency *c*_*i *_of the class with *i *mutations in the generation *t+1 *then calculates as

ci(t+1)=∑j=0iWi−j⋅ci−j(t)⋅Uj⋅e−UW¯⋅j!withW¯=∑i=0∞ci(t)⋅Wi,
 MathType@MTEF@5@5@+=feaafiart1ev1aaatCvAUfKttLearuWrP9MDH5MBPbIqV92AaeXatLxBI9gBaebbnrfifHhDYfgasaacH8akY=wiFfYdH8Gipec8Eeeu0xXdbba9frFj0=OqFfea0dXdd9vqai=hGuQ8kuc9pgc9s8qqaq=dirpe0xb9q8qiLsFr0=vr0=vr0dc8meaabaqaciaacaGaaeqabaqabeGadaaakeaafaqabeqadaaabaGaem4yam2aaSbaaSqaaiabdMgaPbqabaGccqGGOaakcqWG0baDcqGHRaWkcqaIXaqmcqGGPaqkcqWGGaaicqGH9aqpcqWGGaaidaaeWbqaamaalaaabaGaem4vaC1aaSbaaSqaaiabdMgaPjabgkHiTiabdQgaQbqabaGccqGHflY1cqWGJbWydaWgaaWcbaGaemyAaKMaeyOeI0IaemOAaOgabeaakiabcIcaOiabdsha0jabcMcaPiabgwSixlabdwfavnaaCaaaleqabaGaemOAaOgaaOGaeyyXICTaemyzau2aaWbaaSqabeaacqGHsislcqWGvbqvaaaakeaadaqdaaqaaiabdEfaxbaacqWGGaaicqGHflY1cqWGQbGAcqGGHaqiaaaaleaacqWGQbGAcqGH9aqpcqaIWaamaeaacqWGPbqAa0GaeyyeIuoaaOqaaiabbEha3jabbMgaPjabbsha0jabbIgaObqaamaanaaabaGaem4vaCfaaiabdccaGiabg2da9iabdccaGmaaqahabaGaem4yam2aaSbaaSqaaiabdMgaPbqabaGccqGGOaakcqWG0baDcqGGPaqkcqGHflY1cqWGxbWvdaWgaaWcbaGaemyAaKgabeaaaeaacqWGPbqAcqGH9aqpcqaIWaamaeaacqGHEisPa0GaeyyeIuoaaaGccqGGSaalaaa@7BC0@

where *W*_*i*-*j *_is the fitness of individuals with *i-j *mutations. If *c*_*i*_*(t+1) = c*_*i*_*(t) *for all *i*, the population has reached a stable state between mutation pressure and selection against deleterious mutations. The fitness of such a population in mutation-selection balance, relative to a mutation free population, is then W¯
 MathType@MTEF@5@5@+=feaafiart1ev1aaatCvAUfKttLearuWrP9MDH5MBPbIqV92AaeXatLxBI9gBaebbnrfifHhDYfgasaacH8akY=wiFfYdH8Gipec8Eeeu0xXdbba9frFj0=OqFfea0dXdd9vqai=hGuQ8kuc9pgc9s8qqaq=dirpe0xb9q8qiLsFr0=vr0=vr0dc8meaabaqaciaacaGaaeqabaqabeGadaaakeaadaqdaaqaaiabdEfaxbaaaaa@2DF4@.

#### Sexual populations

For the sexual populations we add an additional stage to the asexual model. The individuals in the population are first subject to mutation and selection, following equation (1), and then, subsequent to recombination, are used as a source to form the male and female gamete pool for the next generation. Individuals of the next generation are formed by randomly combining gametes from the latter pool. Infinite sexual populations can be modeled if we replace *c*_*i*_*(t+1) *by *c'*_*i*_*(t) *in equation 1. The term *c'*_*i*_*(t) *now describes the frequencies of mutation classes after the new mutations in the same generation have occurred, and *c*_*i*_*(t) *the distribution before the acquisition of new mutations. If we apply the modified equation 1 separately for both sexes with different values for *U*, we will get two distributions, *c'*_*Ξ*_*i(t) *and *c'*_*X*_*i(t)*, for all *i*. For convenience, we assume here that genomes recombine freely and that none of the mutations occur in homozygous state (i.e. mutations do not coincide at the same locus). An individual bearing a total of *i* mutations on its entire genome can produce gametes containing *0 to i *mutations. We model gamete production as a Bernoulli trials process with *i *chance experiments, with the probability of passing on a mutant allele for each of the *i *heterozygous loci being 1/2. Hence the relative frequencies *p*_*i*_*(k) *of gametes containing *k *mutations produced by an individual with *i *mutations follows a binomial distribution with

pi(k)=(ik)⋅0.5k⋅(1−0.5)i−k=(ik)⋅0.5i.
 MathType@MTEF@5@5@+=feaafiart1ev1aaatCvAUfKttLearuWrP9MDH5MBPbIqV92AaeXatLxBI9gBaebbnrfifHhDYfgasaacH8akY=wiFfYdH8Gipec8Eeeu0xXdbba9frFj0=OqFfea0dXdd9vqai=hGuQ8kuc9pgc9s8qqaq=dirpe0xb9q8qiLsFr0=vr0=vr0dc8meaabaqaciaacaGaaeqabaqabeGadaaakeaacqWGWbaCdaWgaaWcbaGaemyAaKgabeaakiabcIcaOiabdUgaRjabcMcaPiabg2da9maabmaabaqbaeqabiqaaaqaaiabdMgaPbqaaiabdUgaRbaaaiaawIcacaGLPaaacqGHflY1cqaIWaamcqGGUaGlcqaI1aqndaahaaWcbeqaaiabdUgaRbaakiabgwSixlabcIcaOiabigdaXiabgkHiTiabicdaWiabc6caUiabiwda1iabcMcaPmaaCaaaleqabaGaemyAaKMaeyOeI0Iaem4AaSgaaOGaeyypa0ZaaeWaaeaafaqabeGabaaabaGaemyAaKgabaGaem4AaSgaaaGaayjkaiaawMcaaiabgwSixlabicdaWiabc6caUiabiwda1maaCaaaleqabaGaemyAaKgaaOGaeiOla4caaa@5818@

If we pool all male gametes and all female gametes produced by the entire population, the mutation class distribution functions at time *t *for the male gametes *m*_*t*_*(g)*, and for the female gametes *f*_*t*_*(g)*, calculate as:

mt(g)=∑j=g∞c'X(t)⋅pj(g)andft(g)=∑j=g∞c'Ξ(t)⋅pj(g),
 MathType@MTEF@5@5@+=feaafiart1ev1aaatCvAUfKttLearuWrP9MDH5MBPbIqV92AaeXatLxBI9gBaebbnrfifHhDYfgasaacH8akY=wiFfYdH8Gipec8Eeeu0xXdbba9frFj0=OqFfea0dXdd9vqai=hGuQ8kuc9pgc9s8qqaq=dirpe0xb9q8qiLsFr0=vr0=vr0dc8meaabaqaciaacaGaaeqabaqabeGadaaakeaafaqabeqadaaabaGaemyBa02aaSbaaSqaaiabdsha0bqabaGccqGGOaakcqWGNbWzcqGGPaqkcqWGGaaicqGH9aqpcqWGGaaidaaeWbqaaiabdogaJjabcEcaNmaaBaaaleaacqWGybawaeqaaOGaeiikaGcaleaacqWGQbGAcqGH9aqpcqWGNbWzaeaacqGHEisPa0GaeyyeIuoakiabdsha0jabcMcaPiabgwSixlabdchaWnaaBaaaleaacqWGQbGAaeqaaOGaeiikaGIaem4zaCMaeiykaKcabaGaeeyyaeMaeeOBa4MaeeizaqgabaGaemOzay2aaSbaaSqaaiabdsha0bqabaGccqGGOaakcqWGNbWzcqGGPaqkcqWGGaaicqGH9aqpcqWGGaaidaaeWbqaaiabdogaJjabcEcaNmaaBaaaleaacqqHEoawaeqaaaqaaiabdQgaQjabg2da9iabdEgaNbqaaiabg6HiLcqdcqGHris5aOGaeiikaGIaemiDaqNaeiykaKIaeyyXICTaemiCaa3aaSbaaSqaaiabdQgaQbqabaGccqGGOaakcqWGNbWzcqGGPaqkaaGaeiilaWcaaa@700E@

where *g *indicates the number of mutations in the specific gamete class.

By randomly combining gametes from the two distributions in equation 2, we can build the new generation of individuals. The frequencies of the male and female classes with *i *mutations in the new generation before mutation accumulation then calculate as

cΞi(t+1)=cXi(t+1)=∑j=0imt(j)⋅ft(i−j).
 MathType@MTEF@5@5@+=feaafiart1ev1aaatCvAUfKttLearuWrP9MDH5MBPbIqV92AaeXatLxBI9gBaebbnrfifHhDYfgasaacH8akY=wiFfYdH8Gipec8Eeeu0xXdbba9frFj0=OqFfea0dXdd9vqai=hGuQ8kuc9pgc9s8qqaq=dirpe0xb9q8qiLsFr0=vr0=vr0dc8meaabaqaciaacaGaaeqabaqabeGadaaakeaacqWGJbWydaWgaaWcbaGaeuONdGLaemyAaKgabeaakiabcIcaOiabdsha0jabgUcaRiabigdaXiabcMcaPiabg2da9iabdogaJnaaBaaaleaacqWGybawcqWGPbqAaeqaaOGaeiikaGIaemiDaqNaey4kaSIaeGymaeJaeiykaKIaeyypa0ZaaabCaeaacqWGTbqBdaWgaaWcbaGaemiDaqhabeaakiabcIcaOiabdQgaQjabcMcaPiabdccaGiabgwSixlabdccaGiabdAgaMnaaBaaaleaacqWG0baDaeqaaOGaeiikaGIaemyAaKMaeyOeI0IaemOAaOMaeiykaKcaleaacqWGQbGAcqGH9aqpcqaIWaamaeaacqWGPbqAa0GaeyyeIuoakiabc6caUaaa@5B43@

Using *c*_*Ξi*_*(t+1) *and *c*_*Xi*_*(t+1) *again in equation 1 the values for *c'*_*Ξi*_*(t+1) *and *c'*_*Xi*_*(t+1) *can be calculated with the respective mutation rates.

#### Hybridogenetic populations

If a hybrid acquires *z *new mutations in the time span between birth and reproduction, *0 *to *z *of these mutations may end up on the clonally transmitted part of the genome. The probability *q*_*z*_*(x) *that *x *of these *z *mutations end up on the clonally transmitted part of the genome follows again a binomial distribution with qz(x)=(zx)⋅0.5z
 MathType@MTEF@5@5@+=feaafiart1ev1aaatCvAUfKttLearuWrP9MDH5MBPbIqV92AaeXatLxBI9gBaebbnrfifHhDYfgasaacH8akY=wiFfYdH8Gipec8Eeeu0xXdbba9frFj0=OqFfea0dXdd9vqai=hGuQ8kuc9pgc9s8qqaq=dirpe0xb9q8qiLsFr0=vr0=vr0dc8meaabaqaciaacaGaaeqabaqabeGadaaakeaacqWGXbqCdaWgaaWcbaGaemOEaOhabeaakiabcIcaOiabdIha4jabcMcaPiabg2da9maabmaabaqbaeqabiqaaaqaaiabdQha6bqaaiabdIha4baaaiaawIcacaGLPaaacqGHflY1cqaIWaamcqGGUaGlcqaI1aqndaahaaWcbeqaaiabdQha6baaaaa@3F45@.

Using again equation 1, the frequency *f*_*n*_(*t+1*) of eggs containing *n *mutations at time *t+1 *produced by the hybridogens can be calculated as

fn(t+1)=∑y=0n∑i=n∞∑j=n-yi-yWi−j⋅ci−j(t)⋅Uj⋅e−UW¯⋅j!⋅fy(t)⋅mi−j−yci−j(t)⋅qj(n−y)
 MathType@MTEF@5@5@+=feaafiart1ev1aaatCvAUfKttLearuWrP9MDH5MBPbIqV92AaeXatLxBI9gBaebbnrfifHhDYfgasaacH8akY=wiFfYdH8Gipec8Eeeu0xXdbba9frFj0=OqFfea0dXdd9vqai=hGuQ8kuc9pgc9s8qqaq=dirpe0xb9q8qiLsFr0=vr0=vr0dc8meaabaqaciaacaGaaeqabaqabeGadaaakeaacqWGMbGzdaWgaaWcbaGaemOBa4gabeaakiabcIcaOiabdsha0jabgUcaRiabigdaXiabcMcaPiabg2da9maaqahabaWaaabCaeaadaaeWbqaamaalaaabaGaem4vaC1aaSbaaSqaaiabdMgaPjabgkHiTiabdQgaQbqabaGccqGHflY1cqWGJbWydaWgaaWcbaGaemyAaKMaeyOeI0IaemOAaOgabeaakiabcIcaOiabdsha0jabcMcaPiabgwSixlabdwfavnaaCaaaleqabaGaemOAaOgaaOGaeyyXICTaemyzau2aaWbaaSqabeaacqGHsislcqWGvbqvaaaakeaadaqdaaqaaiabdEfaxbaacqWGGaaicqGHflY1cqWGQbGAcqGGHaqiaaGaeyyXIC9aaSaaaeaacqWGMbGzdaWgaaWcbaGaemyEaKhabeaakiabcIcaOiabdsha0jabcMcaPiabgwSixlabd2gaTnaaBaaaleaacqWGPbqAcqGHsislcqWGQbGAcqGHsislcqWG5bqEaeqaaaGcbaGaem4yam2aaSbaaSqaaiabdMgaPjabgkHiTiabdQgaQbqabaGccqWGOaakcqWG0baDcqWGPaqkaaaaleaacqWGQbGAcqGH9aqpcqWGUbGBcqWGTaqlcqWG5bqEaeaacqWGPbqAcqWGTaqlcqWG5bqEa0GaeyyeIuoaaSqaaiabdMgaPjabg2da9iabd6gaUbqaaiabg6HiLcqdcqGHris5aaWcbaGaemyEaKNaemiiaaIaeyypa0JaeGimaaJaemiiaacabaGaemOBa4ganiabggHiLdGccqGHflY1cqWGXbqCdaWgaaWcbaGaemOAaOgabeaakiabcIcaOiabd6gaUjabgkHiTiabdMha5jabcMcaPaaa@98AC@

with W¯=∑i=0∞ci(t)⋅Wi
 MathType@MTEF@5@5@+=feaafiart1ev1aaatCvAUfKttLearuWrP9MDH5MBPbIqV92AaeXatLxBI9gBaebbnrfifHhDYfgasaacH8akY=wiFfYdH8Gipec8Eeeu0xXdbba9frFj0=OqFfea0dXdd9vqai=hGuQ8kuc9pgc9s8qqaq=dirpe0xb9q8qiLsFr0=vr0=vr0dc8meaabaqaciaacaGaaeqabaqabeGadaaakeaadaqdaaqaaiabdEfaxbaacqGH9aqpdaaeWbqaaiabdogaJnaaBaaaleaacqWGPbqAaeqaaOGaeiikaGIaemiDaqNaeiykaKcaleaacqWGPbqAcqGH9aqpcqaIWaamaeaacqGHEisPa0GaeyyeIuoakiabgwSixlabdEfaxnaaBaaaleaacqWGPbqAaeqaaaaa@4111@ and *U *being the genomic mutation rate for the hybrid females. The first term in equation (4) comes from equation (1), delineating mutation and selection. The second term gives the frequency of individuals with *i-j *mutations, having *y *mutations on the egg and *i-j-y *mutations on the sperm. The third term gives the probability that of the *j *mutations occurring in an individual of class *c*_*i-j*_, *n-y *of those mutations (where *n-y *≤ *j*) occur on an egg with *y *mutations to give an egg with *n *mutations. We assume that the distribution of mutation classes within sperms *m*_*l *_is constant over time since the sperms originate from males of the sexual population in mutation selection balance.

The new generation of hybridogens is then again built by randomly combining eggs with sperms. Thus the frequency *c*_*i *_of hybrids with *i *mutations in the next generation before mutation accumulation is again ci(t+1)=∑j=0ifj(t+1)⋅mi−j
 MathType@MTEF@5@5@+=feaafiart1ev1aaatCvAUfKttLearuWrP9MDH5MBPbIqV92AaeXatLxBI9gBaebbnrfifHhDYfgasaacH8akY=wiFfYdH8Gipec8Eeeu0xXdbba9frFj0=OqFfea0dXdd9vqai=hGuQ8kuc9pgc9s8qqaq=dirpe0xb9q8qiLsFr0=vr0=vr0dc8meaabaqaciaacaGaaeqabaqabeGadaaakeaacqWGJbWydaWgaaWcbaGaemyAaKgabeaakiabcIcaOiabdsha0jabgUcaRiabigdaXiabcMcaPiabg2da9maaqahabaGaemOzay2aaSbaaSqaaiabdQgaQbqabaGccqGGOaakcqWG0baDcqGHRaWkcqaIXaqmcqGGPaqkcqGHflY1cqWGTbqBdaWgaaWcbaGaemyAaKMaeyOeI0IaemOAaOgabeaaaeaacqWGQbGAcqGH9aqpcqaIWaamaeaacqWGPbqAa0GaeyyeIuoaaaa@4BC5@.

### (iii) Simulations for finite populations

For the finite populations, we used a Monte Carlo approach to simulate the effect of stochasticity and to be able to model effects of genetic drift and Muller's ratchet. A total of 1000 loci susceptible to deleterious mutations is assumed for all individuals of the different reproductive types. We further assume that initially all genomes are free of deleterious mutations and that back-mutations do not occur. All simulations were programmed in Pascal or C++ using Metrowerks CodeWarrior 5 on an IBM-compatible PC.

#### Asexual populations

At the end of one generation all offspring produced by the individuals are pooled. The offspring inherits all mutations (the inherited and the newly acquired ones) from its parent. The relative contribution of an individual's offspring to this pool corresponds to its fitness compared to the fitness of the other individuals. The fitness is determined by the number of deleterious mutations on an individual's genome and the chosen mutation interaction for the simulation (independent interaction, quadratic interaction or truncation interaction). From this pool of offspring, random individuals are drawn to build the new generation until a preset maximum population size is reached.

#### Sexual populations

In sexual populations, simulation starts with randomly choosing two parents. The likelihood of a parent to sire offspring is proportional to its fitness compared to the rest of the population of the same sex. Once a pair is determined, it produces a single offspring. For convenience, we assume that all mutations in the parent's genome reside on separate chromosomes. The offspring then inherits each of its parent's heterozygous mutations with a probability of 0.5. All homozygous mutations present in the parents are transferred to the offspring. After the process, the individuals are put back into the pool of parents. The whole process is repeated until the maximum population size is reached. After the new generation is established, each individual undergoes mutation accumulation. Note that males and females can have different average mutation rates and that the number of new mutations per individuals follows again a Poisson distribution with the sex specific genomic mutation rate as the mean.

#### Hybridogenetic populations

The simulation starts with a hybridogenetic subpopulation where no mutations are present on the clonally transmitted part of the hybrid's genome (control runs with a starting condition of highly loaded clonal parts have produced the same end results). The sympatric sexual population on the other hand shows the stable distribution of mutation classes, determined with the model for infinite sexual populations. The reproductive cycle starts by randomly combining sperms from males from the sexual population and eggs from the all female hybrid population. As in the previous models, the all-female hybrids acquire a random number of mutations with a probability that follows a Poisson distribution with the mean of the female specific *U*. A newly acquired mutation occurs with the same probability on the hybrid parents' part of the genome as on the sexual parent's part. The fitness of a hybrid is determined by the total number of mutations in its soma (i.e. the number of deleterious mutations on the sexual parent's part of its genome plus the number of mutations on the asexual parent's part). All mutations affect the hybrid's fitness equally, regardless where they reside. At reproduction, the number of gametes produced by the hybrids corresponds to their relative fitness compared to the other hybrids but the gametes contain only the mutations that resided on the clonally transmitted part of the parents' genome. For the next generation random gametes from this pool are drawn and combined with sperms from the infinite sexual population. The frequencies of mutation classes in the sexual parent population remain constant.

### (iv) Testing conditions

Wherever applicable, the same parameter sets were used as in the model of Redfield [19]. Genomic deleterious mutation rate of females was 0.3 per generation whereas the male mutation rate was varied between 0.3, 0.6, 1.8 and 3.0 corresponding to α = 1, 2, 6 and 10. For all reproduction modes, all possible combinations between mutation interaction, population size and male to female mutation rate ratio were tested. Mutation accumulation was followed for 8000 generations and the resulting average fitness for the population calculated. Losses of mutation classes and fixations of mutations were recorded separately. All tests for finite populations were repeated ten times, the results averaged and the standard deviation recorded, since all finite population models are probabilistic.

We furthermore tested the average health of the clonally transmitted part of the genomes of hybridogenetically reproducing populations. This was achieved by calculating the mutation load of a hypothetical diploid population built by combining the clonally transmitted haploid sets of the hybrids with the formula W¯=∑i=0∞[fi∗∑j=0inj∗ni−j]
 MathType@MTEF@5@5@+=feaafiart1ev1aaatCvAUfKttLearuWrP9MDH5MBPbIqV92AaeXatLxBI9gBaebbnrfifHhDYfgasaacH8akY=wiFfYdH8Gipec8Eeeu0xXdbba9frFj0=OqFfea0dXdd9vqai=hGuQ8kuc9pgc9s8qqaq=dirpe0xb9q8qiLsFr0=vr0=vr0dc8meaabaqaciaacaGaaeqabaqabeGadaaakeaadaqdaaqaaiabdEfaxbaacqGH9aqpdaaeWbqaamaadmaabaGaemOzay2aaSbaaSqaaiabdMgaPbqabaGccqGHxiIkdaaeWbqaaiabd6gaUnaaBaaaleaacqWGQbGAaeqaaOGaey4fIOIaemOBa42aaSbaaSqaaiabdMgaPjabgkHiTiabdQgaQbqabaaabaGaemOAaOMaeyypa0JaeGimaadabaGaemyAaKganiabggHiLdaakiaawUfacaGLDbaaaSqaaiabdMgaPjabg2da9iabicdaWaqaaiabg6HiLcqdcqGHris5aaaa@4BCD@. Here, *fi *is the fitness of the class with *i *mutations and *n*_*j *_the frequency of the clonally transmitted haploid set with *j *mutations in the hybrid population.

## Authors' contributions

CS came up with idea for this study, developed the model and wrote the initial version of the manuscript as part of his PhD thesis. H-UR wrote the research proposal that lead to this study, obtained the funding and drafted the present version of the paper. HB helped in rewriting the present version of the manuscript. All authors read and approved of the final manuscript.
